# A Study of the Importance of Clonus Symptoms in Patients with Tramadol Poisoning

**DOI:** 10.1155/2017/2151536

**Published:** 2017-10-11

**Authors:** Fares Najari, Anahita Alizadeh-Ghamsari, Maryam Vahabzadeh, Bita Dadpour, Seyed Reza Mousavi, Ideh Baradaran Kayal

**Affiliations:** ^1^Department of Forensic Medicine and Toxicology, Shahid Beheshti University of Medical Sciences, Tehran, Iran; ^2^Department of Medical Toxicology, Imam Reza Hospital, Mashhad University of Medical Sciences, Mashhad, Iran; ^3^Legal Medicine Organization of Iran, Alborz, Iran

## Abstract

**Objectives:**

Clinical studies to reduce the side effects of tramadol, such as seizure, are necessary. Owing to the high prevalence of tramadol consumption and subsequent complications that result from seizures, the aim of the present study was to find a relationship between clonus and prediction of seizure outcome in patients with tramadol overdose. This can be used to determine the need for essential actions if a significant indicator of preventive medical measures is observed.

**Methods:**

In this case-control study, three groups of patients poisoned with tramadol and with marked ankle clonus were evaluated. A sample size of 50 patients per group was calculated using the Cohen first method. The data were analyzed using SPSS16 software.

**Results:**

All patients with ankle clonus were evaluated. Seizures occurred most commonly in patients aged 21–25 years or younger. The patients who received the preventive medication of magnesium sulfate were seizure-free for 72 h after admission.

**Conclusion:**

It is recommended that, for all patients referred with tramadol poisoning who have symptoms of ankle clonus, the administration of magnesium sulfate should be considered in addition to medication for the prevention of seizures and arrhythmias.

## 1. Introduction

Previous studies have shown that approximately 2–5% of the population will experience at least one nonfebrile seizure during their lifetime [1]. Physicians are responsible for the treatment and termination of seizures, as well as the prevention of complications; they must also determine serious causes, decide the disposition, and reduce the morbidity and mortality associated with this condition. Seizures are a serious complication that may result from medication or drug use and are responsible for nearly 2% of deaths [2].

Tramadol-induced seizures are reported to be generalized tonic-clonic in nature and without auras or focal symptoms, consistent with the observations of the present study. A recent cross-sectional study examined 106 patients who experienced seizures after the ingestion of tramadol [3, 4].

Tramadol is metabolized in the liver by N- and O-didemethylation via the cytochrome P450 pathway, followed by conjugation, and finally excreted by the kidneys. The active metabolite, O-desmethyltramadol (M1), is largely responsible for the analgesic effects of tramadol, as well as the toxic effects, as it has a significantly greater affinity for the central opioid receptors than the parent drug; at doses greater than 800 mg, coma and respiratory depression may occur [5]. Seizures are more common with tramadol than with other opioids and may occur at doses as low as 200 mg. The “serotonin syndrome” is another recognized side effect of tramadol. This syndrome includes the nonspecific symptoms of agitation, tachycardia, confusion, and hypertension [6, 7].

To date, no clinical study of tramadol-induced clonus is available, although there have been similar studies within the field.

A study in Poursina Hospital, Rasht, Iran, showed that, in 68.9% of patients who experienced their first seizure, 21.9% had a recent history of tramadol consumption. The incidence of seizure after tramadol consumption was more prevalent in the initial 10 days and within 6 h of consumption; 73.8% of seizures that resulted from tramadol consumption also occurred during this time. The daily dose of tramadol should not exceed 400 mg. In this cross-sectional study, the mean total dose of tramadol consumption over 24 h was 140.17 ± 73.53 mg [8].

The aim of this study was to identify a relationship between clonus and the prediction of seizure outcome in patients with tramadol overdose. As Imam Reza (AS) hospital in Mashhad, Iran, is one of the referral hospitals for the treatment of poisoned patients, data obtained from this center can be extrapolated to other parts of the country.

## 2. Subjects and Methods

This case-control study was performed on patients with acute tramadol poisoning referred to the Medical Toxicology Center, Imam Reza (p) Hospital, Mashhad, Iran, between March 2013 and March 2015. In all cases, tramadol use was illicit. Data extraction was performed by three independent, trained residents to study the desired objectives by using a specifically developed questionnaire. The quality of data abstraction, “accuracy,” and “completeness” were ensured by the attending pediatrician on several occasions. All authors adhered to the Helsinki recommendations and maintained confidentiality of patient data.

The minimum required sample size of 50 patients for each of the three groups was calculated using the Cohen formula and an odds ratio test with following formula:

(1) $$\begin{array}{c} n={\left(\;\frac{z_{\alpha /2}\sigma }{E}\;\right)}^{2}. \end{array}$$

*z*_*α*/2_ was calculated as 1/99 if *α* is considered 0.05, *E* means error in estimate and equals 0/5, and *σ* means variance.

All hospitalized patients with a history of tramadol overdose were divided into three groups.

Group A: these were patients with acute tramadol poisoning and ankle clonus with no disease, who did not receive any treatment for the prevention of possible seizures.

Group B: these were patients with acute tramadol poisoning and ankle clonus without a specific underlying disease, who received medication (diazepam) for the prevention of seizures.

Group C: these were patients with acute tramadol poisoning and ankle clonus without a specific underlying disease, who received 2 g magnesium sulfate every 8 h for the prevention of seizures.

### 2.1. Inclusion Criteria Were as Follows

1. Patients with marked ankle clonus
2. Positive history of tramadol consumption above 200 mg in addition to positive test results for the qualitative measurement of the urine levels of tramadol
3. No history of mental illness and addiction, as well as no unusual signs in a physical examination
4. Normal anatomical examination, biochemical, and electrolyte test results.

### 2.2. Exclusion Criteria Were as Follows

1. Lack of ankle clonus in patients with acute tramadol poisoning
2. Any specific disorder (such as epilepsy and cerebrovascular attack) in past medical history, history of addiction to drugs or stimulants, and certain diseases
3. Negative test results for the qualitative measurement of urine levels of tramadol
4. Any abnormality in blood gas, electrolyte, and biochemical analyses.

Ankle clonus was defined as the plantar reflection of rhythmic movements in response to a sudden stretch held in muscles and sustained clonus was defined as the presence of five or more beat means of ankle clonus [9]. Based on the Declaration of Helsinki and approved by the respective university ethics committee, informed written consent and clearance were obtained from either the patients or their relatives. All information received from the patients was kept confidential.

### 2.3. Statistical Analysis

Data were descriptively analyzed using SPSS version 21 software. Qualitative data are reported as frequencies and percentages, and quantitative data are reported as mean ± standard deviation. In this study, the confidence interval (CI) was 99%, and a *P* value of <0.01 indicated statistical significance.

## 3. Results

Patients in all three groups had an average dose of consumption (400 ± 200 mg), and no significant differences were observed between dose of tramadol and seizures (*P* > 0.01). None of the patients in the present study had a history of tramadol consumption; all had abused tramadol for the first time. In Group A, 100% of patients had seizures in the first 24 h; however, three patients in Group B had seizures in the second 24 h and seven had seizures in the first 24 h.

Based on inclusion criteria, number of patients in each group was set at 50: 50% were female and 50% were male. The average, view, and median age of the patients were 22 (range: 16–35), 24, and 23 years, respectively. Most of the patients in each group were aged between 20 and 25 years (Table 1), and no significant difference was observed between the sexes in any group (*P* > 0.01). In Group A—that is, patients who did not receive any preventive medication for seizure, but had ankle clonus—37 patients were prone to tonic-clonic seizures (*P* < 0.003); these seizures required treatment, and five deaths occurred as a result of hypoxic anoxic encephalopathy. In Group B, of the patients who had a received maximum of 30 mg of diazepam for seizure prevention, only 10 had tonic-clonic seizures (*P* < 0.01), and all of them were discharged without complications. Among all 50 patients in Group C, who were given 2 g magnesium sulfate every 8 h, none of them experienced seizures, and all of them were discharged after 72 h.

In Groups A and B, all patients with tonic-clonic seizures were younger than 30 years The largest age difference among patients with epilepsy was 23 ± 5 years in all three groups (Table 3). The number of patients with tachycardia (heart rate greater than 100 beats per minute, normal heart rate = 100–60 beats per minute) and loss of consciousness was equal in three groups, but no significant difference was found among the groups regarding heart rate or consciousness and convulsions (*P* > 0.07). In this study, assessing the relationship between ankle clonus and the incidence of seizures in patients under treatment with tramadol, we found that the number of seizures in patients who had not received preventive medicine was 74% (99% confidence interval (CI): 73–75%) and 20% in the group treated with diazepam (99% CI: 19–21%); the mortality rate in this group was 10% (99% CI: 9–11%) (Table 2).

Additionally, patients in all three groups had an average dose of consumption (400 ± 200 mg), and no significant differences were observed between dose of tramadol and seizures (*P* > 0.01). None of the patients in the present study had a history of tramadol consumption; all had abused tramadol for the first time. In Group A, 100% of patients had seizures in the first 24 h; however, three patients in Group B had seizures in the second 24 h and seven had seizures in the first 24 h.

## 4. Discussion

There are some studies showing that clonus can be a sign of CNS excitability [9]; that is, Hidler and Rymer hypothesized the coexistence of both conditions for the occurrence of clonus: reflex pathway delay (involving distal extremity muscles, displaying slow twitch properties) and increasing motor neuron excitability (decrease in motor neuron excitability threshold). These two phenomena disrupt the stability of motor neurons [10].

Convulsions were not observed among patients who were given magnesium sulfate as a membrane stabilizer, which indicated that the impairments caused by tramadol, even at therapeutic doses, affected the membrane potential of brain cells. In addition, 20% of the seizures could be attributed to diazepam among patients treated for the prevention of seizures (valium implied that the full effect of tramadol was to control all seizures). However, further studies are required in this regard. To the best of our knowledge, no study has reported tramadol-induced clonus in the medical literature. In the present study, the preventive effects of two drugs, diazepam and magnesium sulfate, were compared, and the results revealed that the prevention of convulsions in Group B (diazepam) and Group C (magnesium sulfate) was 20% (99% CI: 19–21%) and 100%, respectively. It was found that the anticipated rate of seizure in the two groups did not overlap, and the difference between the two groups was significant (*P* < 0.01), a finding that has not been reported in any of the previous studies [5, 6].

Seizures were highly prevalent in younger patients (younger than 25 years), which can be due to specific genetic variations that can make an individual prone to seizures, as well as pharmacological properties of the drug. In this study, it was shown that the prevalence of drug seizures was not related to the dose and that the majority of patients (over 73%) had abused the drug, with doses of 200 ± 100 mg, which was consistent with other studies [7, 8]. All patients in Group A had a seizure within the first 24 h after consumption, whereas 14% of patients in Group B had seizures in the second 24 h. These findings are consistent with previous studies [11, 12]. In this study, which was performed for the first time in the country, seizures were observed in 47 patients who had marked clonus ankle. Such a finding has not been reported in any of the previous studies, and only the relationship between seizure and ECG changes and pupil size has been evaluated [12, 13]. In a study by Shadnia et al., the dose was 1650 mg, and in a study by Asadi et al., the total dose in the 12 h before seizure was 363.2 ± 303.1 mg (50–1500) [11, 12]. In another study, 57 patients (mean age, 22.3 years; range: 16–43 years; 47 men) were included. Tonic-clonic seizures occurred in 31 (54.4%) patients (26 men and 5 women): single in 14 (45%) patients and multiple in 17 (55%) patients after a tramadol dose in the range of 250–2500 mg. Seizures occurred within 24 h after tramadol intoxication in 26 (84%) patients and later in 5 (16%) patients. In a comparison with addicts without seizures [13, 14] in the same study, tramadol overdose accounted for 1.2% of all poisoning cases (*n* = 158), of which 65% were tramadol only. This study was predominantly conducted in men (63%), and the mean (SD) age was 22.6 (7.4) years. Among the patients, 24 (15%) experienced seizure, death occurred in one patient, and eight cases were treated for potential serotonin syndrome. The risk of seizure increased 2- to 6-fold among users when adjusted for selected comorbidities and concomitant drugs. The risk was the highest among patients aged 25–54 years and those with more than four tramadol prescriptions [4]. In another study, there were 71 cases of tramadol overdose; the seizures were dose related and occurred in eight patients, and there were no cases of serotonin toxicity meeting the Hunter Serotonin Toxicity Criteria [15].

### 4.1. Limitations

One of the limitations of this study was the limited access to some of the required data. Moreover, the serum levels of tramadol were not evaluated, and therefore the correlation with seizure incidence could not be determined. Furthermore, ankle clonus was the only neurological sign that we examined.

## 5. Conclusion

The results of this study suggested that ankle clonus was one of the warning signs and a predicting factor of seizure in patients prescribed tramadol. Therefore, for all patients with tramadol side effects and ankle clonus in their physical examination, the administration of magnesium sulfate is recommended as a cell membrane stabilizer and preventive agent of complications; thus, seizures and possibly death may be prevented.

## Figures and Tables

**Table 1:** The age range of patients poisoned with tramadol referred to Imam Reza (AS) Hospital in Mashhad.

Percentage of patients	Number of patients	Age range
32	16	16–20
50	25	21–25
12	6	26–30
6	3	31–35

**Table 2:** Tonic-clonic seizure in patients of each group, referred to Imam Reza (AS) Hospital in Mashhad.

	Percentage of patients	CI 99%	*P* value
Group A	74	73–75	<0.003
Group B	20	19–21	<0.01
Group C	0	0	

**Table 3:** The frequency of tonic-clonic seizure in patients treated with tramadol with symptoms of ankle clonus, according to their age, referred to Imam Reza (AS) Hospital in Mashhad.

Patients in Group C	Patients in Group B	Patients in Group A	Age range
Zero	(6%) 3	(30%) 15	16–20
Zero	(10%) 5	(34%) 17	21–25
Zero	(4%) 2	(10%) 5	26–30
Zero	Zero	Zero	31–35
